# Attenuation of Aging-Related Oxidative Stress Pathways by Phytonutrients: A Computational Systems Biology Analysis

**DOI:** 10.3390/nu15173762

**Published:** 2023-08-28

**Authors:** V. A. Shiva Ayyadurai, Prabhakar Deonikar

**Affiliations:** Systems Biology Group, CytoSolve Research Division, CytoSolve, Cambridge, MA 02138, USA; prabhakar@cytosolve.com

**Keywords:** oxidative stress, aging, reactive oxygen species, antioxidant enzymes, computational systems biology, CytoSolve, dietary supplements

## Abstract

Aging results from gradual accumulation of damage to the cellular functions caused by biochemical processes such as oxidative stress, inflammation-driven prolonged cellular senescence state, immune system malfunction, psychological stress, and epigenetic changes due to exposure to environmental toxins. Plant-derived bioactive molecules have been shown to ameliorate the damage from oxidative stress. This research seeks to uncover the mechanisms of action of how phytochemicals from fruit/berry/vegetable (FBV) juice powder mitigate oxidative stress. The study uses a computational systems biology approach to (1) identify biomolecular pathways of oxidative stress; (2) identify phytochemicals from FBV juice powder and their specific action on oxidative stress mechanisms; and (3) quantitatively estimate the effects of FBV juice powder bioactive compounds on oxidative stress. The compounds in FBV affected two oxidative stress molecular pathways: (1) reactive oxygen species (ROS) production and (2) antioxidant enzyme production. Six bioactive compounds including cyanidin, delphinidin, ellagic acid, kaempherol, malvidin, and rutin in FBV significantly lowered production of ROS and increased the production of antioxidant enzymes such as catalase, heme oxygenase-1, superoxide dismutase, and glutathione peroxidase. FBV juice powder provides a combination of bioactive compounds that attenuate aging by affecting multiple pathways of oxidative stress.

## 1. Introduction

Advances in science and healthcare over the last century have led to increased life expectancy in humans; however, this extension of lifespan is not without its shortcomings [1,2,3]. As one lives longer, incidence of pathologies such as the development of cardio-metabolic diseases, musculoskeletal diseases, and cancer become more prevalent [4,5,6]. The process of aging results from the accumulation of damages to intra-cellular and inter-cellular functions including metabolic, mitochondrial, genetic, and endolysosomal functions [7,8,9]. The biological processes of cellular aging include oxidative stress, inflammation, immune system malfunction, psychological stress, and epigenetic changes due to exposure to environmental toxins. Oxidative stress is not only a main driver of aging, but also an accelerator of the comorbidities of aging such as osteoarthritis, cardiovascular diseases, diabetes, cancer, and neurological disorders [10,11,12,13]. The secretion of pro-inflammatory cytokines shifts the cell into a senescence-associated secretory phenotype that is implicated in the development of aging and aging-associated comorbidities such as chronic kidney disease and acute kidney injury [14]. 

**Figure 1:**
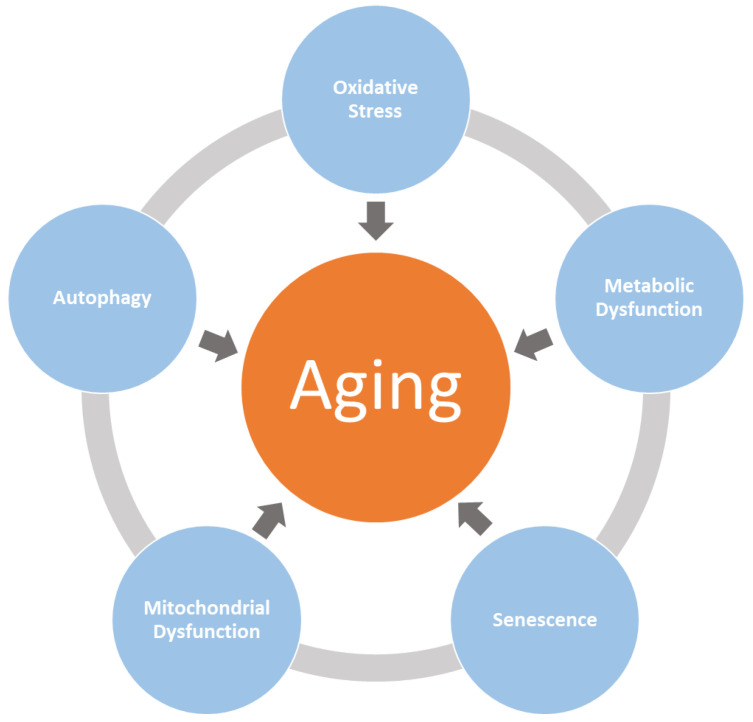
Systems biology of aging. Aging progression is driven by multiple complex biological processes such as oxidative stress, mitochondrial dysfunction, senescence, metabolic dysregulation, and autophagy, leading to accumulation of cellular damages.

Aging is driven by complex mechanisms spanning a multitude of organ systems. These mechanisms fall into five major molecular systems: (1) oxidative stress, (2) mitochondrial dysfunction, (3) senescence, (4) metabolic dysregulation, and (5) autophagy [7,15] that lead to cellular accumulation of damages, as shown in Figure 1. In order to understand the interactions of these systems with one another and their causal relationships with the progression of aging and aging-related comorbidities, a systems biology approach is needed [15]. The scope of this research is to study the progression of aging and aging-related comorbidities using a computational systems biology approach, exploring oxidative stress as a use case.

Oxidative stress is a critical biological phenomenon resulting from an imbalance of the two following processes: (1) the production of reactive oxygen species (ROS) due to either mitochondrial respiration or over-activation of pro-oxidant enzymes like NADPH oxidase (NOX) and (2) the clearance of ROS by cellular antioxidant enzymes such as superoxide dismutase (SOD), heme oxygenase-1 (HO-1), catalase (CAT), and glutathione peroxidase (GPx) [16,17,18,19]. When produced in low to moderate quantities, ROS are necessary to launch a robust immune response from the phagocytes [20] and are also implicated in signaling transduction pathways in endothelial cells, fibroblasts, cardiac myocytes, etc. [21]. However, ROS are highly reactive entities due to their free radical form resulting from having unpaired electron(s) [22], and when produced in excess, ROS damage several key macromolecules including DNA, lipids, and proteins [16,23]. Telomeres are also particularly susceptible to oxidative damage, which accelerates the aging process [24]. Oxidative stress causes mitochondrial dysfunction, loss of antioxidant defenses, loss of integrity of cellular membranes, dysfunction in cellular and DNA repair mechanisms, loss of telomeres, and impaired metabolic regulation, leading to aging and aging-related comorbidities [22,25].

Aging and aging-related comorbidities can be addressed via the mitigation of oxidative stress [26]. Effects of diet and supplementation of plant-based nutrients on the mitigation of oxidative stress have been intensely investigated in human and animal models [27,28,29]. Epidemiological studies have reported that people consuming diets such as the Mediterranean diet, as well as “Blue Zone” diets that emphasize plant-based and lean protein foods rich in antioxidants and anti-inflammatory compounds, undergo healthy aging and have lower incidence of aging-related comorbidities [27,28,29,30]. Vitamins A, D, and E, minerals such as selenium, and phytochemicals such as anthocyanins and flavonoids effectively mitigate oxidative stress by neutralizing ROS and the upregulation of antioxidant enzymes and contribute to anti-aging actions [31,32,33,34,35,36].

Antioxidant effects of phytonutrients in fruits, berries, and vegetables (FBV) has been well-documented in in vivo and in vitro research [37]. Supplementation with FBV dehydrated juice concentrates increases the bioavailability of antioxidant molecules such as polyphenolic compounds [38,39,40] and vitamins [37]. When analyzed for its efficacy on the mitigation of oxidative stress, FBV juice powder reduced DNA damage in lymphocytes [41], as well as ROS levels [42], demonstrating a major role for FBV juice powder phytonutrients in the mitigation of oxidative stress. 

The clinical and experimental data on the effects of FBV juice powder demonstrate ample empirical evidence of its positive effects [43,44]. However, what still remains poorly understood are the mechanistic and quantitative effects of FBV juice powder phytonutrients on aging. There is a need to uncover these complex molecular interactions to develop such understanding that is difficult to ascertain using conventional in vitro and in vivo methods. Computational systems biology and bioinformatics methodologies such as CytoSolve^®^ are being used as a viable tool not only to understand complex biological systems but also to create predictive and quantitative models of these biological systems [45,46,47,48,49,50]. Several studies have established the viability of this methodology to mathematically model biological systems [46,48,50,51,52,53]. In particular, Ayyadurai et al., 2022 used CytoSolve to uncover the mechanisms of action of FBV juice powder phytonutrients on mitigating low-grade chronic inflammation [54]. 

In this study, CytoSolve is used in the following ways: (1) to identify biomolecular pathways of oxidative stress; (2) to identify phytochemicals from FBV juice powder and their specific action on oxidative stress mechanisms; and (3) to quantitatively estimate the effects of FBV juice powder bioactive compounds on oxidative stress. 

## 2. Methods

The methodology used to identify the mechanisms of action of both ROS and antioxidant enzyme production, as well as to quantitatively predict the effects of bioactive compounds from FBV juice powder, involves the use of the well-established CytoSolve protocol [51,52,55], described in detail in Ayyadurai and Deonikar, 2022 [56]. The Supplementary Materials herein provide a detailed summary of the CytoSolve protocol. 

### 2.1. Systematic Review of Literature 

**Figure 2:**
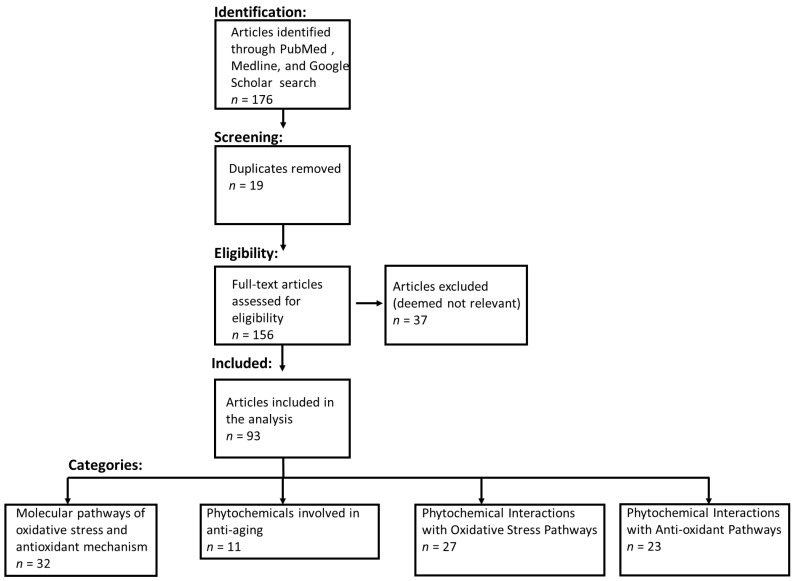
PRISMA flow diagram. Databases such as Medline, PubMed, and Google Scholar were used to identify relevant literature. After removing duplicates, article titles and abstracts were reviewed to identify most relevant articles that contained MeSH keywords and deemed eligible for further comprehensive review.

CytoSolve protocol, as described previously [51,52,55], was employed to identify, organized, curate, review, and extract relevant information from scientific literature. Medical Subject Headings (MeSH) keywords used to obtain relevant scientific literature are provided in Table S4. Per PRISMA guidelines [57], keywords from Table S4 from Section S3 in the Supplementary Materials were used to retrieve the relevant articles for this study, as shown in Figure 2.

### 2.2. CytoSolve In Silico Modeling Protocol

The eligible articles from the systematic literature review process were used to identify and extract biochemical reaction, reaction kinetics, and pharmacokinetic data related to bioactive compounds from FBV juice powder interactions with the molecular pathways of oxidative stress using CytoSolve^®^ protocol [51,52,55,56]. The biochemical reactions, initial concentrations of biochemical parameters involved in these biochemical reactions, and the kinetic parameters used to mathematically model the oxidative stress pathways are documented below. Individual mathematical models are derived from molecular pathways of oxidative stress, and these individual models are integrated to simulate oxidative stress pathways using the standardized CytoSolve^®^ protocol [51,52,55,56]. 

#### 2.2.1. Control Conditions

Under control conditions, the dose level of FBV juice powder is set to zero. ROS and antioxidant enzyme production pathway models involved in aging are simulated, and the concentrations of their respective biomarkers—ROS, catalase, HO-1, SOD, and GPx—are estimated in absence of FBV juice powder supplementation. To understand the effect of FBV juice powder on these biomarkers, a comparison is made between the values of the five biomarkers under control conditions and in the presence of FBV juice powder.

For the control condition, for either the ROS production or the antioxidant enzyme production, the in silico—computational—models assume that the cell is undergoing aging via the oxidative stress state where the NADPH activity, which results in ROS production, is found to increase by approximately two-fold [58]. 

The details of the in silico models used to assess the effects of FBV juice powder phytonutrients on oxidative stress are given below. Two in silico models were constructed to represent oxidative stress signaling transduction pathways. For each of the in silico models, the initial conditions of the parameters, biochemical reactions, the corresponding rate equations, and the kinetic rate constants are provided below.

#### 2.2.2. ROS Production In Silico Model—Initial Conditions, Reactions, Reaction Parameters 

Table 1 contains the information used to model the ROS production pathways implicated in oxidative stress which lead to the formation of ROS. The biochemical reactions and the rate equations used in the in silico model of ROS production are provided in Table S2.1 of Section S2 in Supplementary Materials. The chemical kinetic parameters used in the in silico model of ROS production are provided in Table S2.2 of Section S2 in Supplementary Materials.

#### 2.2.3. Antioxidant Production In Silico Model—Initial Conditions, Reactions, Reaction Parameters

This section contains the information used to model the antioxidant enzyme production pathways involved in oxidative stress. Table 2 contains the information used to model the antioxidant enzyme production pathways involved in oxidative stress that leads to the formation of antioxidant enzymes. The biochemical reactions and the rate equations used in the in silico model of antioxidant enzyme production are provided in Table S3.1 of Section S2 in Supplementary Materials. The chemical kinetic parameters used in the in silico model of antioxidant enzyme production are provided in Table S3.2 of Section S2 in Supplementary Materials.

#### 2.2.4. In Silico—Computational—Analysis of Effect of Bioactive Compounds in FBV Juice Powder on Oxidative Stress Model

Two individual molecular pathway systems in silico models are included in the integrated in silico model: (1) ROS production in silico model and (2) Antioxidant enzyme production in silico model. Individual and combination effects of phytonutrients in FBV juice powder were studied by estimating ROS, catalase, HO-1, SOD, and GPx concentration levels in the presence of FBV juice powder phytonutrients. The standardized CytoSolve^®^ protocol used to simulate the integrated model is detailed previous work [51,52,55,56]. 

Input dosage levels of bioactive compounds are included in Table S1 of the Section S2 in Supplementary Materials. The dose ranges were based on the amounts bioactive compounds found in common fruits, berries, and vegetables [33,65,66,67,68]. The levels of bioactive compounds at the cell surface were based on the serum level for each dose and calculated using their respective Cmax value [69,70,71,72,73,74]. 

The simulation period of seven (7) days was chosen to run the in silico models, since output parameters from all the in silico models attained a steady state within this period. FBV juice powder phytonutrient administration began at the start of the simulations, beginning at t = 0 s, and their levels remained the same for the entire simulation period. 

For the *individual* FBV juice powder phytonutrients, the following in silico simulations were executed:

1. Individual effect of FBV juice powder phytonutrients on ROS;
2. Individual effect of FBV juice powder phytonutrients on CAT;
3. Individual effect of FBV juice powder phytonutrients on HO-1;
4. Individual effect of FBV juice powder phytonutrients on SOD;
5. Individual effect of FBV juice powder phytonutrients on GPx.

For the *combination* of FBV juice powder phytonutrients, the following in silico simulations were executed:

1. Combination effect of FBV juice powder phytonutrients on ROS levels;
2. Combination effect of FBV juice powder phytonutrients on ROS levels on CAT;
3. Combination effect of FBV juice powder phytonutrients on ROS levels on HO-1;
4. Combination effect of FBV juice powder phytonutrients on ROS levels on SOD;
5. Combination effect of FBV juice powder phytonutrients on ROS levels on GPx.

## 3. Results

The systematic bioinformatics literature review yielded an initial set of 176 articles derived by executing fifteen (15) independent searches, as denoted in Table S4, after removing duplicates. The titles and abstracts of these 176 articles were reviewed, and a final set of 93 articles was identified and reviewed comprehensively.

**Figure 3:**
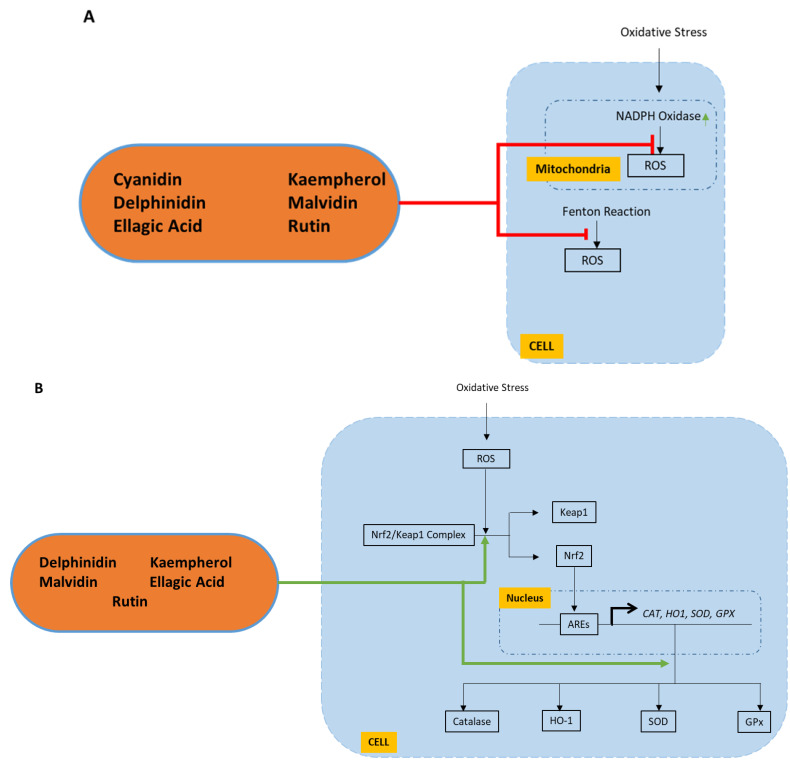
Mechanisms of action of FBV juice powder phytonutrients on oxidative stress pathways. FBV juice powder phytonutrients are enclosed in the oval with blue outline. (**A**) Six (6) FBV juice powder phytonutrients affect one biomarker of ROS production pathway; (**B**) five (5) FBV juice powder phytonutrients affect all four biomarkers of antioxidant enzyme production pathway.

Six FBV juice powder phytonutrients—cyanidin, delphinidin, ellagic acid, kaempherol, malvidin, and rutin—were tested on the in silico model of oxidative stress implicated in aging and showed a significant and measurable effect on both of the oxidative stress molecular pathways. Figure 3 illustrates the interactions of these six FBV juice powder phytonutrients on oxidative stress molecular pathways. The molecular targets from the oxidative stress pathways for the FBV juice powder phytonutrients are detailed in Table 3. 

### 3.1. Effect of FBV Juice Powder Phytonutrients on ROS Production 

**Figure 4:**
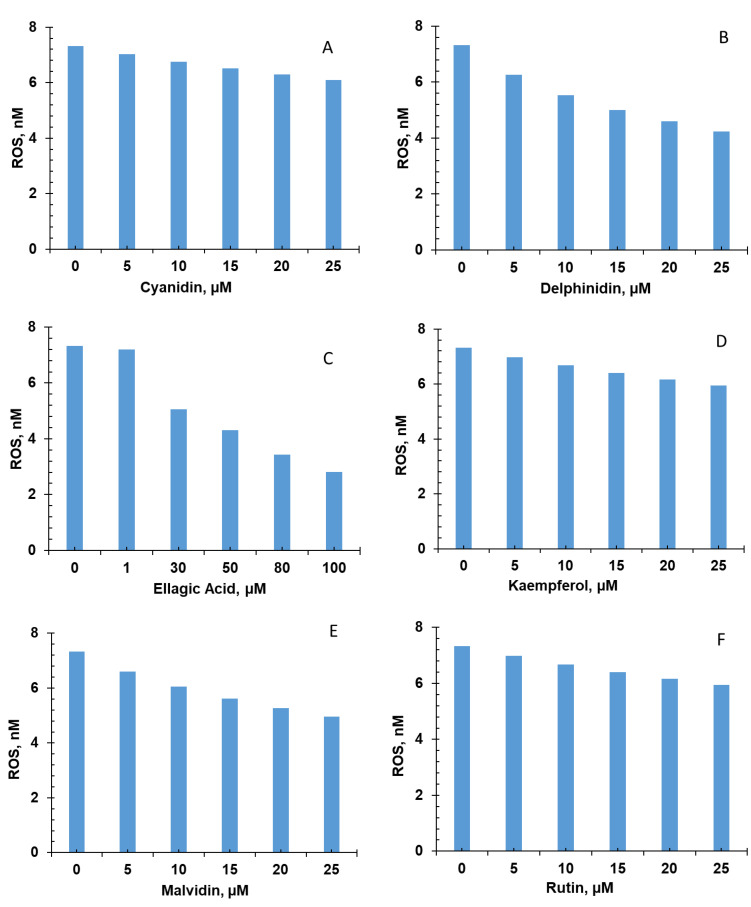

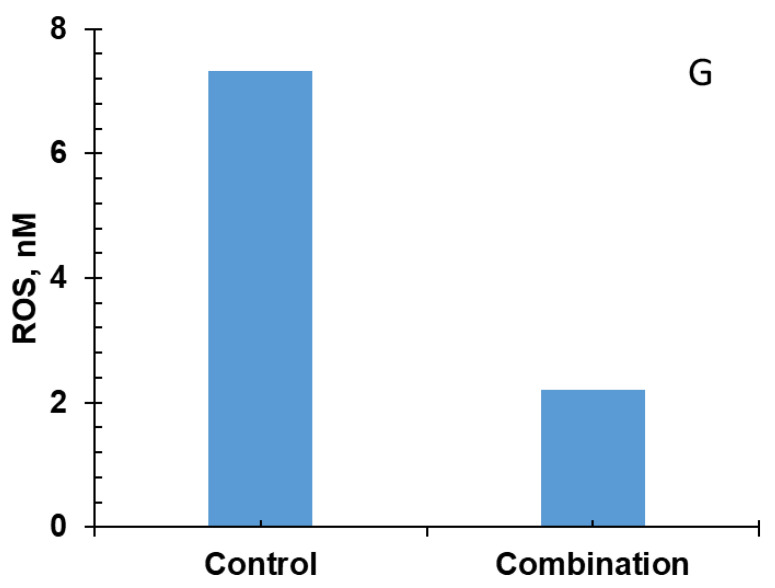
Reactive oxygen species levels in presence of individual and combination of fruit/berry/vegetable juice powder phytonutrients: (**A**) cyanidin, (**B**) delphinidin, (**C**) ellagic acid, (**D**) kampherol, (**E**) malvidin, (**F**) rutin, and (**G**) combination of six FBV juice powder phytonutrients. ROS—reactive oxygen species; FBV—fruit/berry/vegetables.

All six FBV juice powder phytonutrients, including cyanidin, delphinidin, ellagic acid, kaempherol, malvidin, and rutin, targeted the ROS production pathway by scavenging the ROS. The results from individual phytonutrients on ROS production are shown in panels A-F. The effect of the combination of all six phytonutrients is shown in panel G of Figure 4. ROS levels under control conditions are compared with those after administration of FBV powder over a simulation period of 7 days. 

The levels of ROS were estimated to be 7.32 nM under control conditions. Over a 7-day period, ROS concentrations decreased compared to control conditions, as shown in panels A-F of Figure 4, in the presence of the phytonutrients. All six phytonutrients lowered the ROS levels in a dose-dependent manner; however, the highest reductions in ROS levels were observed for delphinidin, ellagic acid, and malvidin, compared to the control. The combination of all the bioactive compounds reduced the ROS levels more than any of the phytonutrient individually (Figure 4, panel G). These results demonstrate the effectiveness of FBV juice powder in attenuating oxidative stress by significantly reducing ROS production. 

### 3.2. Effect of FBV Juice Powder Phytonutrients on Antioxidant Enzyme Production 

**Figure 5:**
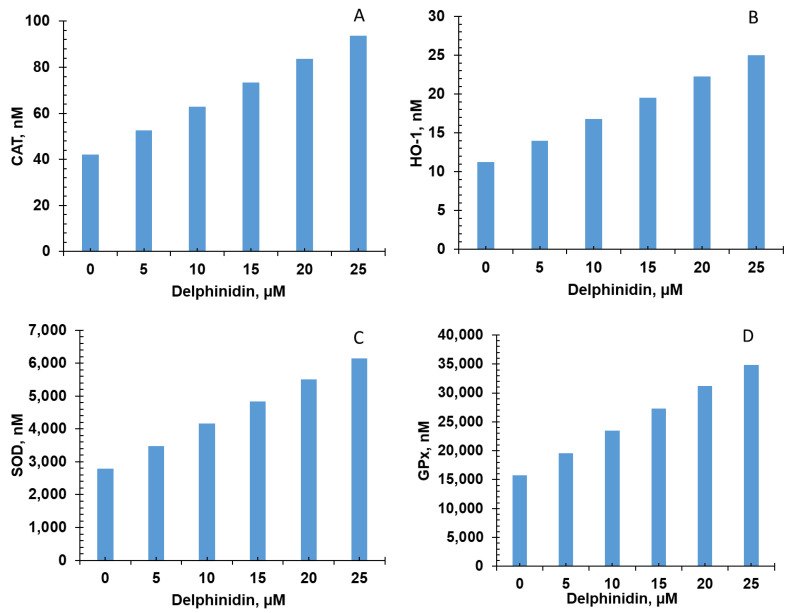
Effect of delphinidin on antioxidant enzyme production over simulation period of 7 days. (**A**) CAT, (**B**) HO-1, (**C**) SOD, and (**D**) GPx production increased dose-dependently with delphinine over a period of 7 days. CAT—Catalase; HO-1—Heme oxygenase; SOD—Superoxide dismutase; GPx—Glutathione peroxidase.

**Figure 6:**
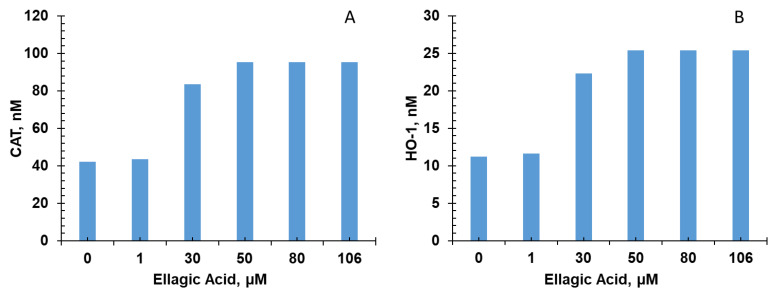

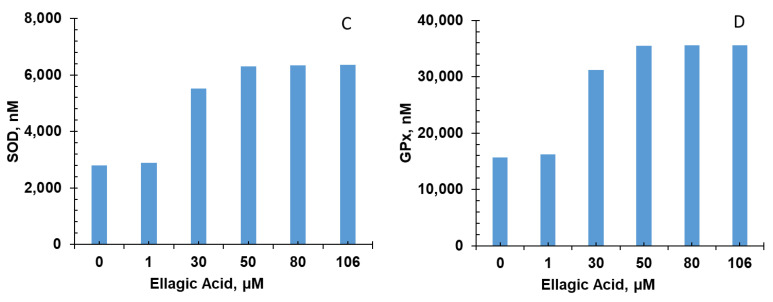
Effect of ellagic acid on antioxidant enzyme production over simulation period of 7 days. (**A**) CAT, (**B**) HO-1, (**C**) SOD, and (**D**) GPx production increased dose-dependently with ellagic acid over a period of 7 days. CAT—Catalase; HO-1—Heme oxygenase; SOD—Superoxide dismutase; GPx—Glutathione peroxidase.

**Figure 7:**
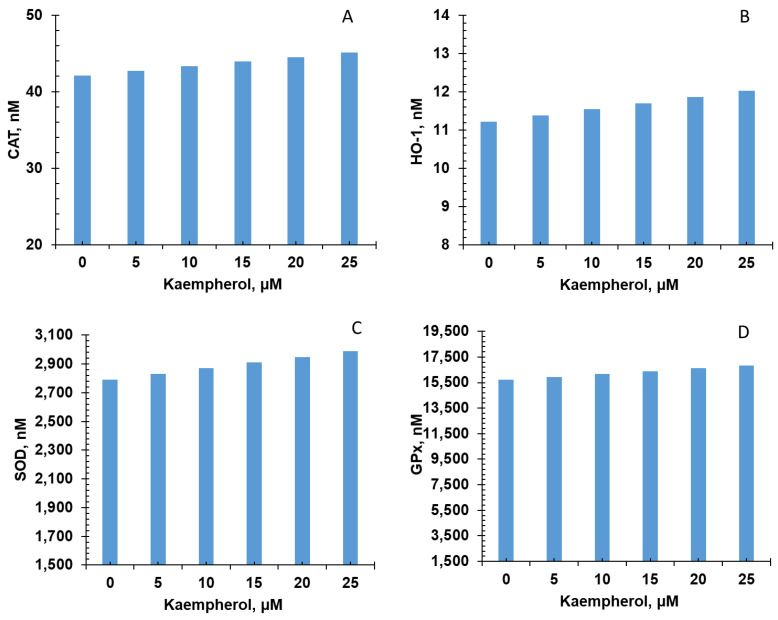
Effect of kaempherol on antioxidant enzyme production over simulation period of 7 days. (**A**) CAT, (**B**) HO-1, (**C**) SOD, and (**D**) GPx production increased dose-dependently with kaempherol over a period of 7 days. CAT—Catalase; HO-1—Heme oxygenase; SOD—Superoxide dismutase; GPx—Glutathione peroxidase.

**Figure 8:**
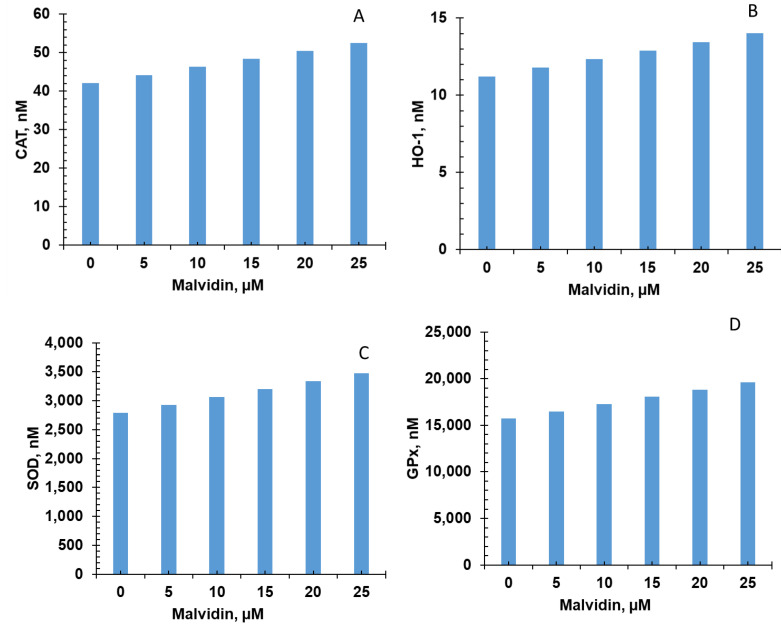
Effect of malvidin on antioxidant enzyme production over simulation period of 7 days. (**A**) CAT, (**B**) HO-1, (**C**) SOD, and (**D**) GPx production increased dose-dependently with malvidin over a period of 7 days. CAT—Catalase; HO-1—Heme oxygenase; SOD—Superoxide dismutase; GPx—Glutathione peroxidase.

**Figure 9:**
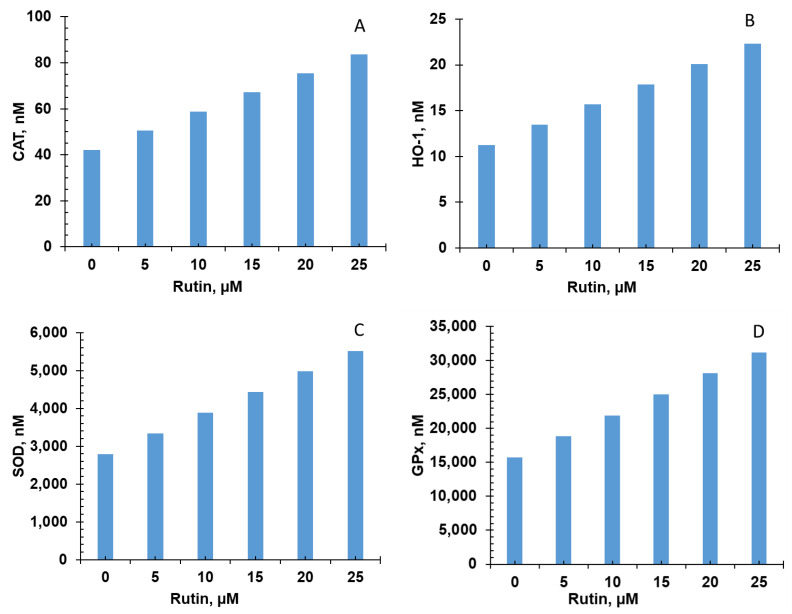
Effect of rutin on antioxidant enzyme production over simulations periods of 7 days. (**A**) CAT, (**B**) HO-1, (**C**) SOD, and (**D**) GPx production increased dose-dependently with rutin over a period of 7 days. CAT—Catalase; HO-1—Heme oxygenase; SOD—Superoxide dismutase; GPx—Glutathione peroxidase.

**Figure 10:**
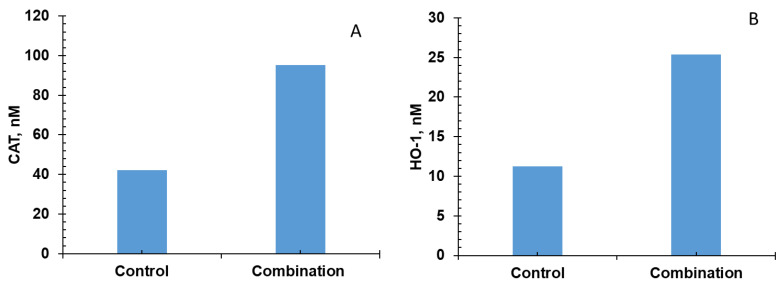

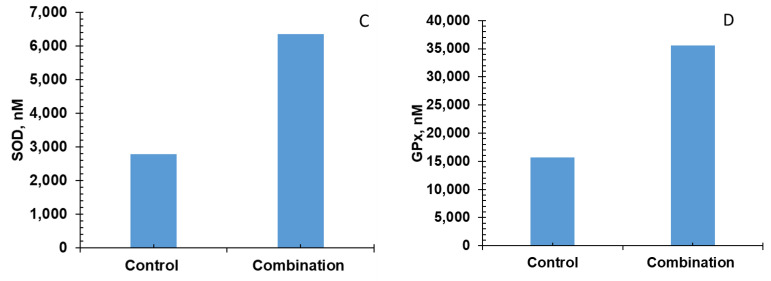
Effect of combination of delphinidin, ellagic acid, kaempherol, malvidin, and rutin on antioxidant enzyme production over simulation period of 7 days. (**A**) CAT, (**B**) HO-1, (**C**) SOD, and (**D**) GPx production increased in presence of all five FBV juice powder phytonutrients combined together over a period of 7 days.

Five of the six bioactive compounds examined in this study from the FBV juice powder—delphinidin, ellagic acid, kaempherol, malvidin, and rutin—targeted the antioxidant enzyme production pathway. The results from individual phytonutrients on antioxidant enzyme production are shown in Figure 5, Figure 6, Figure 7, Figure 8 and Figure 9, and results from the combination of all bioactive compounds are shown in Figure 10. The levels of four antioxidant enzymes—CAT, HO-1, SOD, and GPx—are compared with and without supplementation of FBV juice powder phytonutrients over a period of seven (7) days. 

The concentrations of CAT, HO-1, SOD, and GPx were estimated to be 42.0, 11.2, 2791.0, and 15,718.0 nM, respectively, in the absence of FBV juice powder phytonutrients. All FBV juice powder phytonutrients, except kaempherol, increased the concentrations of all four of the antioxidant enzymes significantly as the dose of phytonutrients increased, as shown in Figure 5, Figure 6, Figure 7, Figure 8 and Figure 9. Supplementation of delphinidin increased the production of all the antioxidant enzymes by 120%. Supplementation of ellagic acid increased the production of all the antioxidant enzymes by 98%; however, increasing the dose of ellagic acid beyond 80 µM did not additionally affect the production of antioxidant enzymes. Supplementation of kaempherol slightly increased the production of all the antioxidant enzymes by only 3%, indicating that the effect of kaempherol on the antioxidant enzyme production pathway was not significant. Supplementation of malvidin increased the production of all the antioxidant enzymes by 25%. Supplementation of rutin increased the production of all the antioxidant enzymes by 40%.

The combination of all the bioactive compounds increased the production of antioxidant enzymes by 128%, as shown in Figure 10. The closest individual compounds that showed such high effect on the production of antioxidant enzymes are ellagic acid and delphinidin. These results demonstrate the effectiveness of FBV juice powder in attenuating oxidative stress by significantly increasing antioxidant enzyme production. 

## 4. Discussion

The scope of this research aimed to provide a novel framework that uses computational systems biology to study the effect of FBV juice powder phytonutrients on the progression of aging and aging-related comorbidities, exploring oxidative stress as a use case. Such a framework is a result of this study, which may be used to discover and develop therapeutic and dietary solutions for aging and aging-related pathologies such as metabolic disorders and cardiovascular disease. This study, a first of its kind to our knowledge, employed a computational systems biology approach, i.e., CytoSolve, to analyze the effect of FBV fruit juice phytonutrients on the molecular pathways of oxidative stress. 

Oxidative stress is a major contributor to aging and aging related comorbidities such as osteoarthritis, cardiovascular diseases, diabetes, cancer, and neurological disorders [85,86,87]. Phytonutrients-rich fruits, berries and vegetables possess antioxidant properties that reduce oxidative stress and thereby may attenuate the aging process [37,88]. The systems biology approach used herein facilitated the understanding the mechanisms of action of such phytonutrients at the molecular level on health and disease progression [89]. Two molecular pathways implicated in oxidative stress are found to be targeted by the six FBV juice powder phytonutrients. The results from this study reveal that after supplementation of FBV juice powder for 7 days, oxidative stress biomarkers such as ROS are downregulated significantly, and the antioxidant enzymes such as CAT, HO-1, SOD, and GPx are upregulated significantly. 

**Figure 11:**
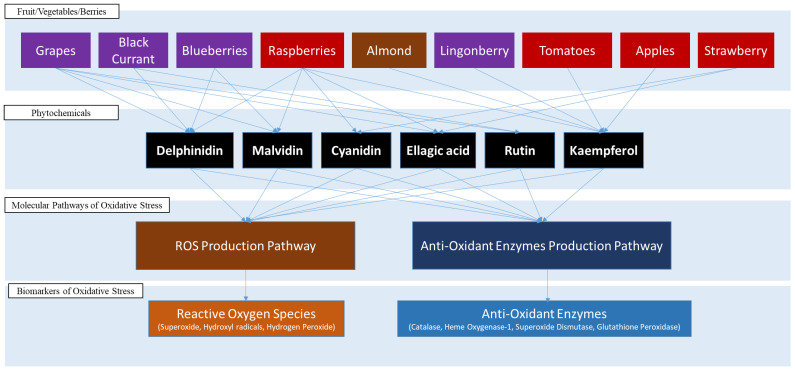
Systems architecture representing FBV juice powder ingredients, phytonutrients, oxidative stress molecular pathways, and biomarkers of oxidative stress.

Based on the results of this study, a four-tiered systems architecture is developed, as shown in Figure 11, to represent the complex interactions between FBV juice powder phytonutrients and the oxidative stress pathways involved in aging. The first tier represents the components of FBV juice powders that contain ingredients such as grapes, black and red currant, blueberries, raspberries, strawberries, lingonberries, almonds, tomatoes, and apples, which have been studied extensively for their antioxidant effects [12,33,67,68,74,75,77,79,80,82,90,91,92]. The second tier consists of the bioactive molecules that are known to interact with the oxidative stress molecular pathways implicated in aging. The two molecular pathway systems—the ROS production pathway and the antioxidant enzyme production pathway—are shown in the third tier. The fourth tier consists of biomarkers of oxidative stress that are directly affected by FBV juice powder phytonutrients. 

Dietary interventional studies have revealed that FBV juice powder supplementation lowered the biomarkers of oxidative stress. Healthy adults supplemented with FBV juice powders showed a marked improvement in biomarkers of oxidative stress such as SOD [93]. A controlled dietary human intervention study reported that fruit and vegetable consumption led to the mitigation of DNA and lipid oxidation due to ROS production [91]. A randomized controlled clinical trial in overweight or obese subjects reported improved antioxidant activity of enzymes such as CAT, GPx, and SOD after supplementation of tomato juice [92] for 20 days. The mitigation of oxidative stress by FBV has been attributed to their potent antioxidant bioactive molecules [33,68]. Experimental studies have shown that bioactive components such as delphinidin, malvidin, cyanidin, ellagic acid, rutin, and kaempherol scavenge ROS effectively [12,67,68,79,83,90]. In addition, delphinidin, malvidin, cyanidin, ellagic acid, rutin, and kaempherol also mitigated oxidative stress by increasing the production and/or activity of antioxidant enzymes CAT, HO-1, SOD, and GPx [68,74,75,80,82]. 

**Figure 12:**
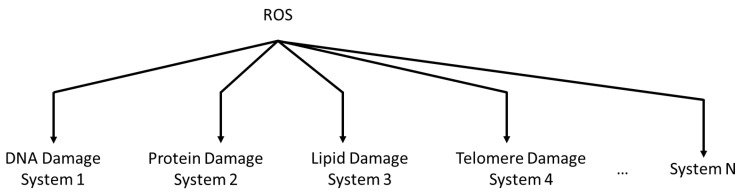
Downstream effect of ROS on multiple molecular systems of aging including DNA damage, protein damage, lipid damage, telomere damage, etc. ROS—Reactive oxygen species.

The in silico computational systems biology analysis herein corroborates the clinical in vivo and experimental in vitro observations discussed above. This study reveals that individual bioactive compounds, namely delphinidin, malvidin, cyanidin, ellagic acid, rutin, and kaempherol, reduce ROS production, as well as upregulate antioxidant enzyme production. Moreover, the quantitative and predictive results herein further reveal that the combination of the phytonutrients, which simulates the consumption of FBV juice powder, performs better than any one individual bioactive compound in lowering ROS and upregulating antioxidant enzymes. ROS may affect many downstream molecular systems of ageing such as the degradation of biomolecules such as DNA, proteins, lipids, telomeres, etc., leading to damage accumulation and acceleration of aging and aging-related morbidities [22,25]. Results from this study indicate that bioactive compounds from FBV juice powder may prevent the degradation of such biomolecules. The existing computational model can be expanded and scaled up to incorporate an *N* number of such molecular systems, as illustrated in Figure 12. 

The computational systems biology approach from this study provides a molecular mechanistic explanation of how the FBV juice powder phytonutrients attenuate oxidative stress, a critical mechanism of aging, which is difficult to determine based on just the clinical and experimental observations.

## 5. Strengths and Limitations of the Study

### 5.1. Strengths

The strengths of this study are discussed as follows. 

To the best of our knowledge, this study is the first of its kind to demonstrate the efficacy of FBV juice powder phytonutrients on oxidative stress quantitatively and mechanistically using a computational systems biology approach. 

The computational biology platform used in the this study—CytoSolve—provides a distributed engineering systems approach that uses a scalable framework to develop models of a large number of molecular pathways representing complex biological phenomenon that incorporates multiple spatial and temporal scales [47]. 

The work conducted in this study for oxidative stress can be further scaled to include other molecular processes such as autophagy, mitochondrial dysfunction, metabolic dysfunction, and senescence—shown in Figure 1—that are involved in the progression of aging and its comorbidities. 

### 5.2. Limitations

The limitations to this study are discussed as follows. 

Although not unique to this study, variation in the parameters used to model oxidative stress computationally arising due to experimental conditions (e.g., cell type, cell culture methods, etc.) can add uncertainty to the results from this study. They are inherent to many computational models of cellular systems [94], and further experimentation is needed to validate such computational models. 

Although the results from this study corroborate clinical [12,67,68,79] and experimental observations [68,74,75,80,82], additional studies will serve to further validate the effect of FBV juice powder phytonutrients on oxidative stress. Such experimental studies are planned in future work.

Finally, the oxidative stress model consists of two molecular pathways: the ROS production pathway and the antioxidant enzyme production pathway. This model can be expanded to integrate relevant downstream pathways that include the effect of ROS on DNA damage, lipid peroxidation, protein damage, and telomere damage, which are critical to the progression of aging.

## 6. Conclusions and Future Work

### 6.1. Conclusions

A computational systems biology model of oxidative stress is developed to analyze the effect of FBV juice powder phytonutrients on five oxidative stress biomarkers—ROS, CAT, HO-1, SOD, and GPx. Six FBV juice powder phytonutrients are identified that individually and when combined together downregulate all five biomarkers of oxidative stress, which is substantiated by clinical and experimental studies. All six phytonutrients—cyanidin, delphinidin, ellagic acid, kaempherol, malvidin, and rutin—lower ROS, whereas only five of them—delphinidin, ellagic acid, kaempherol, malvidin, and rutin—were efficient in upregulating of antioxidant enzyme production. 

The molecular systems architecture of oxidative stress and its interactions with the bioactive molecules of FBV juice powders developed in this study provides a systems-level understanding of complex biological molecular interactions involved in processes such as aging and aging-associated pathologies. The framework developed herein offers mechanistic insights that explain how FBV juice powder phytonutrients mitigate oxidative stress and may affect the process of aging. 

### 6.2. Future Work

This research now provides a framework that uses computational systems biology approach to study the progression of aging and aging related comorbidities beyond oxidative stress. This framework is currently being expanded to explore the other four molecular systems of aging such as mitochondrial dysfunction, senescence, metabolic dysregulation, and autophagy. Additionally, this framework can be expanded to study the mitigating effect of FBV juice powder on aging-related pathologies such as cardiovascular disease, diabetes, osteoarthritis, neurodegenerative disease, etc., as oxidative stress is also critically implicated in these pathologies. The predictive in silico models from this study can be utilized to discover dietary and therapeutic interventions for aging and aging-related comorbidities. 

## Figures and Tables

**Table 1:** Initial concentrations used in ROS production model.

Species	Value (nM)	Ref.
LH	351,000	Babbs and Steiner, 1990 [59]
O_2_	10,000	Atunes et al., 1996 [60]
H_2_O	5.5 × 10^7^	Shi et al., 2013 [61]
Fe^2+^	100.0	Atunes et al., 1996 [60]
Fe^3+^	6800.0	Atunes et al., 1996 [60]
SOD	700.0	Kavdia et al., 2011 [62]
H_2_O_2_	0.38	Atunes et al., 1996 [60]
Catalase	41.03	Aydemir and Kuru, 2003 [63]
GSH	1000	Shi et al., 2013 [61]
GPr	1	Shi et al., 2013 [61]
NADPH Oxidase	3.06 × 10^−4^	Atunes et al., 1996 [60]

LH—Lipid peroxide; SOD—Superoxide dismutase; O_2_—Oxygen; H_2_O—Water; Fe^3+—^Ferric ion; Fe^2+^—Ferrous ion; H_2_O_2_—Hydrogen peroxide; GSH—Glutathione; GPr—Glutathione peroxidase; NADPH—Nicotinamide adenine dinucleotide phosphate.

**Table 2:** Initial concentrations used in anti-oxidant enzymes production model.

Species	Name	Value (nM)	Ref.
Maf	M AF nuclear protein	4000	Khalil et al., 2015 [64]
Keap1	Kelch-like-ECH-associated protein 1	2000	Khalil et al., 2015 [64]
Nrf2	Nuclear factor-erythroid 2 p45-related factor 2	1800	Khalil et al., 2015 [64]
H_2_O_2i_	Basal H_2_O_2_	1500	Khalil et al., 2015 [64]

**Table 3:** Summary of the oxidative stress pathway targets interacting with FBV juice powder phytonutrients.

Bioactive Compound in FBV Juice Powder	Oxidative Stress Pathway Target	Biological Effect	Ref.
(1) Delphinidin	Nrf2	Upregulation of Nrf2 nuclear translocation	J. Xu et al., 2020 [75]
	ROS	Inhibition of ROS production	Jin et al., 2013 [76]
(2) Malvidin	Nrf2	Upregulation of SOD and GPx gene expression	Merecz-Sadowska et al., 2023 [68]; Y. Xu et al., 2021 [77]
	ROS	Neutralization of ROS	Merecz-Sadowska et al., 2023 [68]
(3) Cyanidin	ROS	Neutralization of ROS	Acquaviva et al., 2016 [78]; Chun et al., 2003 [79]
(4) Ellagic acid	Nrf2	Downregulation of Keap1 mRNA and protein expression and upregulation of Nrf2 mRNA and protein expression	Ding et al., 2019 [80]
	ROS	Scavenging of ROS	A. Kumar et al., 2021 [81]
(5) Rutin	Nrf2	Upregulation of the expression of Nrf2 activator p21	Gȩgotek et al., 2017 [82]
	ROS	Scavenging of ROS	Gȩgotek et al., 2017 [82]; Patil et al., 2013 [83]
(6) Kaempferol	Nrf2	Inhibition of NRF2 degradation	A. D. N. Kumar et al., 2016 [74]; Imran et al., 2019 [84]
	ROS	Scavenging of ROS	Rahul et al., 2020

## Data Availability

All relevant data are within the paper and its Supporting Information files.

## Supplementary Materials

The following supporting information can be downloaded at: https://www.mdpi.com/article/10.3390/nu15173762/s1. The Supplementary Materials contains three Sections. Section S1 contains CytoSolve® Operating Guide Protocol Summary. Section S2 contains three tables: Table S1, Table S2.1, Table S2.2, Table S3.1, and Table S3.2. Table S1 contains information about the bioactive components and their concentration range used in this study. Table S2.1 contains the biochemical reactions and rate equations involved in ROS production model. Table S2.2 contains chemical kinetic parameters used in ROS production model. Table S3.1 contains the biochemical reactions and rate equations involved in anti-oxidant production model. Table S3.2 contains chemical kinetic parameters used in anti-oxidant production model. Section S3 contains Table S4 that lists search strings used to identify literature.
