# Supplementary material for: Attenuation of Aging-Related Oxidative Stress Pathways by Phytonutrients: A Computational Systems Biology Analysis
Source: Nutrients. 2023 Aug 28;15(17):3762. doi: 10.3390/nu15173762 (PMC10489992; doi:10.3390/nu15173762)
Supplement: Supplementary file 1 [file nutrients-15-03762-s001.zip › nutrients-2567509-supplementary.pdf]

Attenuation of Aging-related Oxidative Stress Pathways by Phytonutrients: A Computational Systems Biology Analysis

V.A. Shiva Ayyadurai[1*] and Prabhakar Deonikar[1]

Supplementary Information

Section S1

CytoSolve® Operating Guide Protocol Summary

S1.1. Introduction to CytoSolve® System

CytoSolve is a well-established computational systems biology framework of technology and processes that provides the capability to derive molecular mechanisms of action; to create quantitative and predictive models of those mechanisms; and, to employ the resultant models to simulate complex biomolecular phenomena [1–5]. In neurovascular studies, the CytoSolve framework elicited and derived a multi-layered engineering molecular systems architecture integrating the anatomy of the neurovascular unit, molecular mechanisms, and disease to demonstrate the commonality of multiple neurovascular diseases as communication dysfunctions in common molecular signaling sub-systems and compounds [4].

In oncology, CytoSolve's capability has been employed for the *in silico* modeling of pancreatic cancer to identify and optimize a multi-combination therapeutic that was subsequently allowed for clinical trials by the United States Food and Drug Administration [6], has been used to identify the molecular systems architecture of interactome in acute myeloid leukemia (AML) microenvironment [7], and has been independently recognized by leading cancer researchers as a

platform for developing multi-combination therapies [3]. In cardiovascular research, CytoSolve has been used to accurately model the release of nitric oxide (NO) production in endothelial cells subjected to shear stress [5].

In the area of plant biology, CytoSolve enabled the quantitative molecular systems understanding of C1 metabolism - a critical system of molecular pathways inherent to all plants, fungi and bacteria - to understand the systemic effects oxidative stress and genetic modification on C1 metabolism in soy [8–11]. Recently, CytoSolve was used to uncover the mechanistic understanding of glucaric acid effect on liver detoxification [12], identify the synergistic effect of apigenin and hesperidin on joint pain [13], discover and model the mechanisms of immunomodulatory effect of bioactive compound in green tea on organ transplant tolerance [14], and elucidate effect of bioactive compounds from fruit, berry, vegetable (FBV) juice power on low grade chronic inflammation [15].

S1.2. CytoSolve® System Capabilities

The method used in this study provides a scalable computational framework for modeling large-scale biological systems by dynamic integration of an ensemble of multiple molecular pathway models [1]. This method enables the development of large-scale models of complex biological systems that span multiple temporal and spatial scales as well as across diverse domains. Rather than attempting to monolithically model systems of biochemical reactions, a distributed engineering systems approach – a relatively novel concept in systems biology– is employed that breaks a large scale biological system into an ensemble of smaller molecular pathway models that

are computationally coupled. This approach makes the modeling of large-scale biological systems both tractable and scalable.

S1.3. Key Elements of CytoSolve® System Protocol

There are six (6) steps that comprise the protocol to use the CytoSolve® system. Fig. S1 illustrates those steps of the protocol.

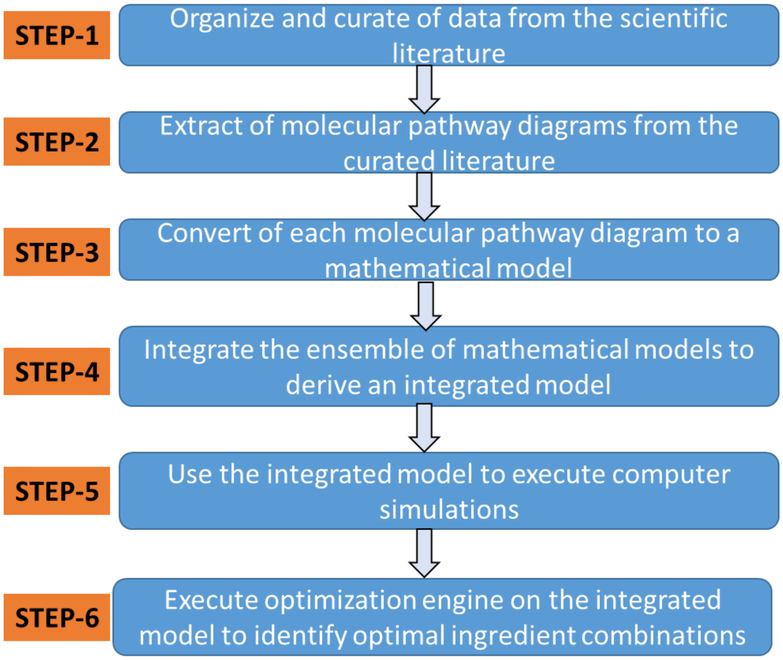

Figure S1: CytoSolve® Protocol Overview. The six steps involved in the CytoSolve® protocol. Steps 1 and 2 relate to performing systematic literature review to identify molecular pathways and the biochemical parameters required for computational modeling. Steps 3 to 5 relate to construction of individual models, integration of individual models, and executing simulations using the integrated modes. Step 6 provides the option to employ CytoSolve® optimization engine to discover the optimal combination of inputs (ingredients/compounds) for a specific range of values of outputs (biomarkers associated with particular biomolecular functions).

The six (6) steps are listed below:

1) Organize and curate data from the scientific literature (§S1.4)

2) Extract molecular pathway diagrams from the curated literature (§S1.5)

3) Convert each molecular pathway diagram to a mathematical model (§S1.6)

4) Integrate individual mathematical models to derive an integrated model (§S1.7)

5) Use the integrated model to execute computer simulations to analyze the effect of ingredients on interest, individually as well as in combination (§S1.8)

6) Execute optimization engine on the integrated model to identify optimal ingredient combination (§S1.9)

S1.4. CytoSolve® Protocol for Organization and Curation of Data from the Scientific Literature

This protocol step is the first step of the overall CytoSolve® Protocol indicated in Fig.S1. In this step, the scientific literature is searched to identify journal papers that contain research on the *area of interest* i.e. oxidative stress, inflammation, apoptosis, joint pain, cardiovascular health, etc. For a particular area of interest, and *ingredients of interest*, molecular pathways for the area of interest, and the effect of ingredients of interest on those molecular pathways are identified and organized.

Four (4) specific steps are executed per the CytoSolve® Protocol to organize and curate the journal papers, as itemized below:

1. Create a list of Medical Subject Headings (MeSH) *keywords* to optimize recall and precision of peer-reviewed articles. The keywords are typically a list of words in combination with Boolean operators i.e. “AND”, “OR”, etc.
2. Search and retrieve the relevant peer-reviewed articles published during a specific *time period* from PubMed, Medline, and Google Scholar. These set of articles are stored as an “Initial Set” repository. The time period is a date range i.e. “January 1989 to April 2022,” etc.

3. Screen the titles and abstracts of articles in the Initial Set repository to identify most relevant articles based on our inclusion criteria. These set of articles are stored as the "Final Set" repository
4. Perform full-length review of peer-reviewed articles from the Final Set repository

Abstracts and unpublished literature were not sought as they have not been peer reviewed adequately to authenticate their results.

Articles obtained from the systematic literature review are categorized into three categories: 1) Articles related to molecular pathways implicated in the area of interest; 2) articles related to interactions of ingredients of interest with molecular targets in area of interest pathways; and, 3) articles related to pharmacokinetic properties of the ingredients of interest.

S1.5. CytoSolve® Protocol for Extraction of Data from the Scientific Literature

Journal articles in Group 1 are reviewed to gather data relevant to molecular pathways involved in the area of interest. The steps to extract and represent molecular pathways diagrammatically are itemized below:

1. Identify and extract:
    a. chemical species involved in the molecular pathways
    b. types of cells
    c. cellular components (e.g. cytosol, mitochondria, nucleus, etc.) where the chemical species are present in each cell type

2. Identify and diagrammatically represent biochemical interactions

3. Interconnect biochemical reactions to create molecular pathway diagram in each cell type

Journal articles in Group 2 are reviewed to gather data relevant to pharmacokinetics of ingredients of interest.

Journal articles in Group 3 are reviewed to extract following information:

1. Reaction rate constants of biochemical reactions involved in molecular pathways
2. Molecular targets of ingredients of interest in the molecular pathways of interest

The kinetic parameters used in this study are derived using principles of Michaelis-Menten kinetics that are based on the steady-state approximation of the biochemical reactions [16,17]. This information is provided in Section S2.

S1.6. CytoSolve® Protocol for Setup of Individual In Silico Mathematical Models

The steps to convert molecular pathway diagrams to mathematical models are itemized below:

1. Convert biochemical reactions involved in each of the molecular pathway into ordinary differential equations (mathematical expressions that describe the rate of change)

2. Represent each molecular pathway as a system of ordinary differential equations

3. Encode the system of differential equations in a computer software source code format known as Systems Biology Markup Language (SBML)[18] to construct a mathematical model for a particular molecular pathway

4. Store each model as a separate SBML file

S1.7. CytoSolve® Protocol for Integration of Individual In Silico Mathematical Models

In order to create an integrative quantitative model of the area of interest, it is necessary to mathematically couple the solutions across the ensemble of individual molecular pathway models. Such mathematical coupling is performed using the CytoSolve [2,14,19] computational engine, which is described in detail in Ayyadurai and Dewey, 2011[1] and Ayyadurai et al., 2022. The computational architecture of CytoSolve enables the integration of plurality of molecular pathway models [1,19].

The steps to integrate the ensemble of individual mathematical models are listed below:

1. Upload individual SBML files, constructed in §S1.6 of Section S1, to CytoSolve engine
2. Update the initial conditions for the molecular species in all the mathematical models in the graphical user interface
3. Update simulation period was specified in the graphical user interface
4. Review and confirm molecular species and reaction duplicates across all the individual models in the graphical user interface
5. Commence integration of individual models

For a computational system biology analysis wherein the simulation of biochemical reactions is being executed and the governing equations are well known, as is in this case, the error bounds are set prior to executing simulations [20,21]. Herein, the error bounds are set to 10^{-6} prior to the execution of simulations. This means that the solutions to the governing equations used in the

simulation of specific biochemical reactions must be within these error bounds. Therefore, *in silico* – computational – results from such simulations will not have error bars, which customarily appear in results reported from *in vitro* and *in vivo* experimental studies [22].

S1.8. CytoSolve® Protocol for Simulation of Integrated In Silico Mathematical Model

The following steps are performed to execute the computer simulations:

1. Input biochemical reactions for interaction between ingredients of interest and molecular pathways involved in area of interest
2. Input the kinetic rate constants for each of the biochemical reaction
3. Input the initial concentrations for each of the molecular species in the biochemical reactions
4. Input the time period for the simulation of integrative models, and dose levels of ingredients of interest
5. Execute the integrative model under control conditions
6. Execute the integrative model in presence of ingredients of interest individually
7. Execute the integrative model in presence of the combination of ingredients

The steps for analyzing simulation output data are itemized below:

1. Export the raw data to Microsoft Excel
2. Extract the steady state levels of biomarkers
3. Plot steady state levels of biomarkers as a function of simulation time in presence and absence of ingredients of interest, individually as well as in combination.

S1.9. CytoSolve® Protocol to Execute Optimization Engine on the Integrated Model to Identify Optimal Ingredient Combination

The following steps are performed to execute the optimization engine:

1. Simulate the integrative model in presence of ingredients of interest
2. Select "Optimize Results" on the CytoSolve® graphical user interface to initiate the optimization engine
3. In the optimization engine, choose "Objective" for the ingredients of interest and the biomarkers affected by the ingredients of interest. The objective is set to "optimize" for the ingredients of interest; "maximize" for the biomarkers that are related to positive outcome; and, "minimize" for the biomarkers that are related to negative outcomes.
4. Execute the optimization engine and record the results

The steps for analyzing simulation output data are itemized below:

1. Export the raw data to Microsoft Excel
2. Extract the values of ingredients of interest that have either minimized or maximized the biomarker

S1.10. CytoSolve® System Constraints

Although the framework developed in this study provides a detailed mechanistic understanding of oxidative stress that match well with published clinical data, some of the model components' parameters were derived from experiments using different cell types, as well as different experimental conditions such as variations in culture conditions that adds to the uncertainty of the

model predictions [23]. Such issues of parameter estimation, however, are not unique to this study. They are common to a number of cellular mathematical models [23] and do warrant further experimental investigation and validation.

Section S2

Table S1: Bioactive components amounts in FBV juice powder dose used for simulation

Bioactive Component	Range of Amount Used for Simulations (g)	Concentrations Used for Simulation (µM)
Cyanidin	0 to 10	0 to 25
Delphinidin	0 to 11	0 to 25
Ellagic acid	0 to 0.85	0 to 106
Kaempherol	0 to 0.1	0 to 25
Malvidin	0 to 39	0 to 25
Rutin	0 to 5	0 to 25

Table S2.1: Biochemical Reactions and Rate Equations Involved in ROS Production.

Description	Rate Equation
$[L^{\cdot}] + [O_2] \rightarrow [LOO^{\cdot}]$	kLPO * $[L^{\cdot}]$ * $[O_2]$
$[LOO^{\cdot}] + [LH] \rightarrow [LOOH] + [L*]$	$[LOO^{\cdot}]$ *[LH]* *kLR1
$[LOOH] + [Fe^{3+}] \rightarrow [LOO^{\cdot}] + [Fe^{2+}] + [H^{+}]$	[LOOH] * $[Fe^{3+}]$ * kLRFe2
$[LOOH] + [Fe^{2+}] \rightarrow [LO^{\cdot}] + [Fe^{3+}] + [OH^{-}]$	[LOOH] * $[Fe^{2+}]$ * kLRFe1
$[H_2O_2] + [Catalase] \rightarrow [H_2O] + [O_2]$	$[H_2O_2]$ * [Catalase] * kcat
$[LH] + [LO^{\cdot}] \rightarrow [LOH] + [L*]$	[LH] * $[LO^{\cdot}]$ * kLR2
$[GPo] + [GSH] \rightarrow [GSGP] + [GSSG] + [H_20]$	[GPo] * [GSH] * kGSGP
$[GSH] + [GSGP] \rightarrow [Gpr] + [GSSG] + [H_20]$	[GSH] * [GSGP] * kGPr
$[H_2O_2] + [GPr] \rightarrow [H2O] + [GPo]$	$[H_2O_2]$ * $[H^{+}]$ * $[H^{+}]$ * [GPr] * kGPo
$[H^{+}] + [O_2^{\cdot-}] + [SOD] \rightarrow [H_2O_2]$	$[H^{+}]$ * $[O_2^{\cdot-}]$ * [SOD] * kSOD
$[H2O] \leftrightarrow [H^{+}] + [OH^{-}]$	[H2O] * kdH2O - $[H^{+}]$ * $[OH^{-}]$ * KH2O
$[LH] + [OH^{\cdot}] \rightarrow [L^{\cdot}] + [H_2O]$	[LH] * $[OH^{\cdot}]$ * kinitLR
$[H_2O_2] + [Fe^{2+}] \rightarrow [Fe^{3+}] + [OH^{\cdot}] + [OH^{-}]$	$[H_2O_2]$ * $[Fe^{2+}]$ * kFe1
$[Fe^{2+}] + [OH^{\cdot}] \rightarrow [OH^{-}] + [Fe^{3+}]$	$[Fe^{2+}]$ *$[OH^{\cdot}]$ * kFe6
$[Fe^{3+}] + [H_2O_2] \rightarrow [Fe^{2+}] + [HO_2^{\cdot}] + [H^{+}]$	kFe5 * $[Fe^{3+}]$ * $[H_2O_2]$
$[HO_2^{\cdot}] + [Fe^{3+}] \rightarrow [O_2] + [Fe^{2+}] + [H^{+}]$	$[HO_2^{\cdot}]$ * $[Fe^{3+}]$ * kFe3
$[O_2^{-*}] + [Fe^{3+}] \rightarrow [Fe^{2+}] + [O_2]$	$[O_2^{-*}]$* $[Fe^{3+}]$ * kFe3
$[H_2O_2] + [OH^{\cdot}] \rightarrow [H O_2*] + [H_2O]$	$[H_2O_2]$ * $[OH^{\cdot}]$ * kFe4
$[HO_2^{\cdot}] + [OH^{\cdot}] \rightarrow [H_2O] + [O_2]$	$[HO_2^{\cdot}]$ * $[OH^{\cdot}]$ * kFe7
$[H O_2^{\cdot}] + [H_2O_2] \rightarrow [OH^{\cdot}] + [H_2O] + [O_2]$	$[H O_2^{\cdot}]$ * $[H_2O_2]$ * kFe9
$[H O_2^{\cdot}] + [HO_2^{\cdot}] \rightarrow [H_2O_2]$	$[H O_2^{\cdot}]$ * $[H O_2^{\cdot}]$ * kFe8
$[O_2] \rightarrow [O_2^{\cdot-}]$	$[O_2]$* (kNADPHOxi * [NADPH OXidase] / ((km_NADPHoxi + O2)
$[O_2^{\cdot-}] \rightarrow [O_2]$	$[O_2^{-*}]$* kdegROS

Table S2.2: Chemical Kinetic Parameters used in ROS Production Model.

Parameter	Value	Units	References
kinitLR	0.5	nM^{-1} s^{-1}	Atunes et al., 1996[24]
kLPO	0.3	nM^{-1} s^{-1}	Atunes et al., 1996[24]
kLR1	1.4×10^{-8}	nM^{-1} s^{-1}	Atunes et al., 1996[24]
kFe1	7.6×10^{-8}	nM^{-1} s^{-1}	Henle et al., 1996[25]
kFe6	0.35	nM^{-1} s^{-1}	Henle et al., 1996[25]
kFe3	3.1×10^{-4}	nM^{-1} s^{-1}	Henle et al., 1996[25]
kLR2	0.0066	nM^{-1} s^{-1}	Atunes et al., 1996[24]
kLRFe1	0.015	nM^{-1} s^{-1}	Xue et al., 2012[26]
kLRFe2	1×10^{-6}	nM^{-1} s^{-1}	Xue et al., 2012[26]
kGPo	0.021	nM^{-1} s^{-1}	Buettner et al., 2006[27]
kGSGP	4×10^{-5}	nM^{-1} s^{-1}	Buettner et al., 2006[27]
kGPr	0.01	nM^{-1} s^{-1}	Buettner et al., 2006[27]
kSOD	1.6	nM^{-1} s^{-1}	Edwards et al., 2011[28]
kcat	0.034	nM^{-1} s^{-1}	Edwards et al., 2011[28]
KH2O	140.0	nM^{-1} s^{-1}	Stillinger, 1978[29]
kdH2O	2.5×10^{-5}	s^{-1}	Stillinger, 1978[29]
kFe2	0.0012	nM^{-1} s^{-1}	Henle et al., 1996[25]
kFe4	0.027	nM^{-1} s^{-1}	Henle et al., 1996[25]
kFe5	2.7×10^{-10}	nM^{-1} s^{-1}	Henle et al., 1996[25]
kFe7	7.0	nM^{-1} s^{-1}	Henle et al., 1996[25]
kFe8	0.017	nM^{-1} s^{-1}	Henle et al., 1996[25]
kFe9	5×10^{-10}	nM^{-1} s^{-1}	Henle et al., 1996[25]
kFe10	5.5	nM^{-1} s^{-1}	Henle et al., 1996[25]
kT_LOO	0.001	nM^{-1} s^{-1}	Xue et al., 2012[26]
kVE_LOO_deg	2×10^{-5}	nM^{-1} s^{-1}	Xue et al., 2012[26]
kBC_O2	1.8×10^{-17}	nM^{-1} s^{-1}	Xue et al., 2012[26]
kBCR_O2	0.2	nM^{-1} s^{-1}	Ozhogina and Kasakina, 1995[30]
kBC_kBCR	8×10^{-7}	nM^{-1} s^{-1}	Ozhogina and Kasakina, 1995[30]
kBC_LOO	3×10^{-6}	nM^{-1} s^{-1}	Ozhogina and Kasakina, 1995[30]
kBC_VITE	10	nM^{-1} s^{-1}	Haila, 1999[31]
kBCO_LOO	1×10^{-4}	nM^{-1} s^{-1}	Haila, 1999[31]
kAsc_OH	0.00855	nM^{-1} s^{-1}	Haila, 1999[31]
kAsc_O2	0.01	nM^{-1} s^{-1}	Haila, 1999[31]
kAScR_O2	0.26	nM^{-1} s^{-1}	Haila, 1999[31]
kIQ_XO	280	nM	Haila, 1999[31]
k32	720	nM s^{-1}	Haila, 1999[31]
kEpi_ROS	0.0073	nM^{-1} s^{-1}	Haila, 1999[31]

kdegROS	0.085	s^{-1}	Macfarlane and Miller, 1992[32]
kNADPHOxi	1720	s^{-1}	Koshkin et al., 1997[33]
km_NADPHoxi	30000	nM	Koshkin et al., 1997[33]
kI_Epi_NDAPhoxi	5000	nM	Estimated
kiEGCG_Fe	0.046	s^{-1}	Perron et al. 2010[34]
ki_lyco_L	0.013	nM^{-1} s^{-1}	Kawata et al., 2018[35]
ki_ly_LOO	0.08	nM^{-1} s^{-1}	Kawata et al., 2018[35]
Ki_ging_O2	4050	nM	Guenette et. al., 2007[36]
KIshog_O2	850	nM	Guenette et. al., 2007[36]
Rutin_SuperoxideIC50	47942	nM	Patil et al., 2013[37]
Malvidin-3-O-glucoside_SuperoxideIC50	21482	nM	Abeda et al. 2015[38]
Kaempherol_SuperoxideIC50	47930	nM	Wang et al. 2018[39]
EllagicAcid_SuperoxideIC50	27660	nM	Sun et al., 2021[40]
Delphinidin3Oglucoside_SuperoxideIC50	13321	nM	Abeda et al. 2015[38]
Cyanidin_SuperoxideIC50	56015	nM	Chun et al., 2003[41]

Table S3.1: Biochemical Reactions and Rate Equations Involved in Anti-Oxidant Production Model.

Description	Rate Equation
[NRF2] + [Keap1] + [AKT] → [NRF2_Keap1]	[Nrf2] * [Keap1] * konNrf2_Keap1 – [Nrf2_keap1] * koffNrf2_Keap1
[NRF2_Keap1] →Φ	[Nrf2_keap1] * kdegNRf2
[NRF2] + [Maf] → [NRF2_Maf]	[Nrf2_N] * [Maf] * knrf2_maf – [Nrf2_Maf] * koffnrf2_maf
[Nrf2_keap1_o] → [Keap1] + [NRF2_N]	[Nrf2_keap1_o] * ktrans_Nrf2
[NRF2-ARE-Maf] → [HO-1 mRNA]	[Nrf2_ARE_Maf] * kHO1mRNA
[HO-1 mRNA] → [HO-1 mRNA_c]	[HO-1_mRNA] * ktrans
[HO-1 mRNA_c] → Φ	[HO-1_mRNA_c] * kdegHO1mRNA
[HO-1 mRNA_c] → [HO-1]	[HO-1_mRNA_c] * ksynHO1 - HO-1 * kdegHO1
[NRF2-ARE-Maf] → [SOD2_mRNA]	[Nrf2_ARE_Maf] * ksyn_SODmRNAsyn
[SOD2 mRNA] → [SOD2 mRNA_c]	[SOD2_mRNA] * ktrans
[SOD2 mRNA_c] → Φ	[SOD2_mRNA_c] * kdeg_SODmRNAdeg
[SOD2 mRNA_c] → [SOD2]	[SOD2_mRNA_c] * ksyn_SOD2protein – [SOD2] * kdeg_SOD2protein
[NRF2-ARE-Maf] → [CAT mRNA]	[Nrf2_ARE_Maf] * ksyn_CATmRNAsyn
[CAT mRNA] → [CAT mRNA_c]	[CAT_mRNA] * ktrans
[CAT mRNA_c] → Φ	[CAT_mRNA_c] * kdeg_CATmRNAdeg
[CAT mRNA_c] → [CAT]	[CAT] * kdeg_CATprotein – [CAT_mRNA_c] * ksyn_CATprotein
[NRF2-ARE-Maf] → [GPx-1 mRNA]	[Nrf2_ARE_Maf] * ksyn_GPXmRNAsyn

[GPx-1 mRNA] → [GPx-1 mRNA_c]	[GPX_mRNA] * ktrans
[GPx-1 mRNA_c] → Φ	[GPX_mRNA_c] * kdeg_GPXmRNAdeg
[GPx-1 mRNA_c] → [GPx-1]	[GPX] * kdeg_GPXprotein – [GPX_mRNA_c] * Ksyn_GPXprotein

Table S3.2: Chemical Kinetic Parameters Used in Anti-Oxidant Production Model.

Parameter	Value	Units	Reference
ktrans	0.005775	s^{-1}	Dargemont and Kuhn 1992[42]
konNrf2_Keap1	0.00345	$nM^{-1}s^{-1}$	Uruno et al. 2013 [43]
koffNrf2_Keap1	0.282	s^{-1}	Uruno et al. 2013 [43]
kdegNRf2	$3.85*10^{-4}$	s^{-1}	Mulvaney et al. 2016 [44]
ktrans_Nrf2	$7.70*10^{-4}$	s^{-1}	Jain et al., 2017 [45]
knrf2_maf	$1.67*10^{-5}$	$nM^{-1}s^{-1}$	Khalil et al. 2015 [43]
koffnrf2_maf	0.167	s^{-1}	Calculated based on Khalil et al. 2015 [43]
k_keap1_ox	$4.0*10^{-5}$	$nM^{-1}s^{-1}$	Khalil et al. 2015 [43]
k_keap1_red	$1.00*10^{-4}$	$nM^{-1}s^{-1}$	Khalil et al. 2015 [43]
ktrans_Nrf22	0.00167	s^{-1}	Khalil et al. 2015[43]
kHO1mRNA	$2.69*10^{-5}$	s^{-1}	Noel et al. 2015 [44]
kdegHO1mRNA	$8.37*10^{-5}$	s^{-1}	Bouton and Demple, 2000 [46]
ksynHO1	$6.32*10^{-5}$	s^{-1}	Ge et al. 2017 [47]
ksyn_SODmRNAsyn	2.61E-05	s^{-1}	Murakami et al., 2017 [48]
ksyn_CATmRNAsyn	8.50E-05	s^{-1}	Murakami et al., 2017 [48]
ksyn_GPXmRNAsyn	5.12E-04	s^{-1}	Murakami et al., 2017 [48]
kdeg_GPXmRNAdeg	5.07E-05	s^{-1}	Shen et al. 1994 [49]
kdeg_SODmRNAdeg	1.20E-05	s^{-1}	Fukai et al. 1999 [50]
kdeg_CATmRNAdeg	5.92E-05	s^{-1}	Sen et al., 2005 [51]
Ksyn_GPXprotein	9.75E-04	s^{-1}	Szychowski et al. 2019 [52]
kdeg_GPXprotein	8.02E-06	s^{-1}	Shen et al. 1994 [49]
ksyn_CATprotein	3.21E-05	s^{-1}	Szychowski et al. 2019 [52]
kdeg_CATprotein	1.45E-05	s^{-1}	Chen et al. 2018 [53]
ksyn_SOD2protein	5.14E-04	s^{-1}	Hass and Massaro 1988 [54]
kdeg_SOD2protein	3.85E-06	s^{-1}	Bartnikas and Gitlin, 2003 [55]
k_aARE	5.24E+27		Ge et al. 2017 [47]
kd_ARE	0.02		Ge et al. 2017 [47]
kDelphinidin3Oglucoside_NRF2EC50	20000	nM	Xu et al. 2020 [56]
kEllagicAcid_NRF2EC50	30000	nM	Ding et al. 2019 [57]
k Kaempherol _NRF2EC50	349369	nM	Kumar et al., 2016 [58]

k Malvidin-3-O-glucoside _NRF2EC50	100,000	nM	Xu et al. 2021 [59]
kRutin_NRF2EC50	25,000	nM	Gęgotek et al., 2017 [60]

Section S3

Table S4. Search strings used to identify literature.

Literature Search Keywords	
1.	"Oxidative stress AND aging"
2.	"Reactive oxygens species production AND oxidative stress"
3.	"Anti-oxidant enzyme production AND oxidative stress"
4.	"Cyanidin AND Reactive oxygens species production"
5.	"Delphinidin AND Reactive oxygens species production"
6.	"Ellagic acid AND Reactive oxygens species production"
7.	"Kaempherol AND Reactive oxygens species production"
8.	"Malvidin AND Reactive oxygens species production"
9.	"Rutin AND Reactive oxygens species production"
10.	"Delphinidin AND Anti-oxidant enzyme production"
11.	"Ellagic acid AND Anti-oxidant enzyme production"
12.	"Kaempherol AND Anti-oxidant enzyme production"
13.	"Malvidin AND Anti-oxidant enzyme production"
14.	"Rutin AND Anti-oxidant enzyme production"

Bibliography

1. Ayyadurai, V.A.S.; Dewey, C.F. CytoSolve: A Scalable Computational Method for Dynamic Integration of Multiple Molecular Pathway Models. *Cell. Mol. Bioeng.* **2011**, *4*, 28–45.
2. Nordsletten, D.A.; Yankama, B.; Umeton, R.; Ayyadurai, V.V.S.; Dewey, C.F. Multiscale Mathematical Modeling to Support Drug Development. *IEEE Trans. Biomed. Eng.* **2011**, *58*, 3508–3512.
3. Al-Lazikani, B.; Banerji, U.; Workman, P. Combinatorial drug therapy for cancer in the post-genomic era. *Nat. Biotechnol.* **2012**, *30*, 679–92.
4. Sweeney, M.D.; Ayyadurai, S.; Zlokovic, B. V. Pericytes of the neurovascular unit: key functions and signaling pathways. *Nat. Neurosci.* **2016**, *19*, 771–783.
5. Koo, A.; Nordsletten, D.; Umeton, R.; Yankama, B.; Ayyadurai, S.; García-Cardeña, G.; Dewey, C.F. In Silico Modeling of Shear-Stress-Induced Nitric Oxide Production in Endothelial Cells through Systems Biology. *Biophys. J.* **2013**, *104*, 2295–2306.
6. Food and Drug Administration Center for Drug Evaluation and Research Request for Determination of Exempt Status of Investigational New Drug Application (IND) for Cyto-001 as Treatment for Patients with Pancreatic Cancer (PIND: 118833) 2013.
7. Ayyadurai, V.A.S.; Deonikar, P.; McLure, K.G.; Sakamoto, K.M. Molecular Systems Architecture of Interactome in the Acute Myeloid Leukemia Microenvironment. *Cancers 2022, Vol. 14, Page 756* **2022**, *14*, 756.
8. Ayyadurai, V.A.S.; Deonikar, P. Do GMOs Accumulate Formaldehyde and Disrupt Molecular Systems Equilibria? Systems Biology May Provide Answers. *Agric. Sci.* **2015**, *06*, 630–662.
9. Kothandaram, S.; Deonikar, P.; Mohan, M.; Venugopal, V.; Ayyadurai, V.A.S. <i>In Silico</i> Modeling of C1 Metabolism. *Am. J. Plant Sci.* **2015**, *06*, 1444–1465.
10. Mohan, M.; Kothandaram, S.; Venugopal, V.; Deonikar, P.; Ayyadurai, V.A.S. Integrative Modeling of Oxidative Stress and C1 Metabolism Reveals Upregulation of Formaldehyde and Downregulation of Glutathione. *Am. J. Plant Sci.* **2015**, *06*, 1527–1542.
11. Ayyadurai, V.A.S.; Hansen, M.; Fagan, J.; Deonikar, P. In-Silico Analysis & In-Vivo Results Concur on Glutathione Depletion in Glyphosate Resistant GMO Soy, Advancing a Systems Biology Framework for Safety Assessment of GMOs. *Am. J. Plant Sci.* **2016**, *07*, 1571–1589.
12. Ayyadurai, V.A.S.; Deonikar, P.; Fields, C. Mechanistic Understanding of D-Glucaric Acid to Support Liver Detoxification Essential to Muscle Health Using a Computational Systems Biology Approach. *Nutrients* **2023**, *15*.
13. Ayyadurai, V.A.S.; Deonikar, P. In Silico Modeling and Quantification of Synergistic Effects of Multi-Combination Compounds: Case Study of the Attenuation of Joint Pain Using a Combination of Phytonutrients. *Appl. Sci.* **2022**, *12*, 10013.
14. Ayyadurai, V.A.S.; Deonikar, P. Bioactive compounds in green tea may improve transplant tolerance: A computational systems biology analysis. *Clin. Nutr. ESPEN* **2021**, *46*.
15. Ayyadurai, V.A.S.; Deonikar, P.; Bannuru, R.R. Attenuation of low-grade chronic inflammation by phytonutrients: A computational systems biology analysis. *Clin. Nutr. ESPEN* **2022**.
16. Cornish-Bowden, A. One hundred years of Michaelis–Menten kinetics. *Perspect. Sci.* **2015**.

17. Michaelis, L.; Menten, M.L.; Goody, R.S.; Johnson, K.A. Die Kinetik der Invertinwirkung/ The kinetics of invertase action. *Biochemistry* **1913**.
18. Hucka, M.; Finney, A.; Sauro, H.M.; Bolouri, H.; Doyle, J.C.; Kitano, H.; Arkin, A.P.; Bornstein, B.J.; Bray, D.; Cornish-Bowden, A.; et al. The systems biology markup language (SBML): A medium for representation and exchange of biochemical network models. *Bioinformatics* **2003**, *19*, 524–531.
19. Ayyadurai, V.A.S. Services-Based Systems Architecture for Modeling the Whole Cell: A Distributed Collaborative Engineering Systems Approach. In; 2010; pp. 115–168.
20. Shmulevich, I.; Aitchison, J.D. DETERMINISTIC AND STOCHASTIC MODELS OF GENETIC REGULATORY NETWORKS. *Methods Enzymol.* **2009**, *467*, 335.
21. Oden, J.T. (John T.; Reddy, J.N. (Junuthula N. *An Introduction to the Mathematical Theory of Finite Elements*; 1976th ed.; Dover Publications: New York, 2011; ISBN 9780486142210.
22. Cumming, G.; Fidler, F.; Vaux, D.L. Error bars in experimental biology. *J. Cell Biol.* **2007**, *177*, 7.
23. Thanh, V.H.; Zunino, R.; Priami, C. Efficient finite-difference method for computing sensitivities of biochemical reactions. *Proc. R. Soc. A Math. Phys. Eng. Sci.* **2018**, *474*.
24. Antunes, F, Salvador, A, Marinho, HS, Alves, R, P.R. Lipid Peroxidation in mitochondrial inner membranes. An integrative mechanistic model. *Free Radic. Biol. Med.* **1996**, *21*, 917–943.
25. Henle, E.S.; Luo, Y.; Linn, S. Fe 2+ , Fe 3+ , and Oxygen React with DNA-Derived Radicals Formed during Iron-Mediated Fenton Reactions †. *Biochemistry* **1996**, *35*, 12212–12219.
26. Xue, C.; Chou, C.-S.; Kao, C.-Y.; Sen, C.K.; Friedman, A. Propagation of cutaneous thermal injury: A mathematical model. *Wound Repair Regen.* **2012**, *20*, 114–122.
27. Buettner, G.R.; Ng, C.F.; Wang, M.; Rodgers, V.G.J.; Schafer, F.Q. A New Paradigm: Manganese Superoxide Dismutase Influences the Production of H2O2 in Cells and Thereby Their Biological State. *Free Radic. Biol. Med.* **2006**, *41*, 1338–1350.
28. Edwards, A.; Cao, C.; Pallone, T.L. Cellular mechanisms underlying nitric oxide-induced vasodilation of descending vasa recta. *Am. J. Physiol. Physiol.* **2011**, *300*, F441–F456.
29. Stillinger, F.H. Proton Transfer Reactions and Kinetics in Water. In *THEORETICAL CHEMISTRY: Advances and Perspectives*; ACADEMIC PRESS, INC., 1978; Vol. 3, pp. 177–234.
30. Ozhogina, O.A.; Kasaikina, O.T. Β-Carotene As an Interceptor of Free Radicals. *Free Radic. Biol. Med.* **1995**, *19*, 575–581.
31. Haila, K. *Effects of Carotenoids and Carotenoid- Tocopherol Interaction on Lipid Oxidation In Vitro*; 1999; Vol. 62; ISBN 9514586670.
32. MacFarlane, N.G.; Miller, D.J. Depression of peak force without altering calcium sensitivity by the superoxide anion in chemically skinned cardiac muscle of rat. *Circ. Res.* **1992**, *70*, 1217–1224.
33. Koshkin, V.; Lotan, O.; Pick, E. Electron transfer in the superoxide-generating NADPH oxidase complex reconstituted in vitro. *Biochim. Biophys. Acta - Bioenerg.* **1997**, *1319*, 139–146.
34. Perron, N.R.; Wang, H.C.; Deguire, S.N.; Jenkins, M.; Lawson, M.; Brumaghim, J.L. Kinetics of iron oxidation upon polyphenol binding. *Dalt. Trans.* **2010**, *39*, 9982–9987.
35. Kawata, A.; Murakami, Y.; Suzuki, S.; Fujisawa, S. Anti-inflammatory Activity of β-

Carotene, Lycopene and Tri-n-butylborane, a Scavenger of Reactive Oxygen Species. *In Vivo (Brooklyn).* **2018**, *32*, 255–264.
36. Guénette, S.A.; Ross, A.; Marier, J.F.; Beaudry, F.; Vachon, P. Pharmacokinetics of eugenol and its effects on thermal hypersensitivity in rats. *Eur. J. Pharmacol.* **2007**, *7*, 60–67.
37. Patil, S.L.; Mallaiah, S.H.; Patil, R.K. Antioxidative and radioprotective potential of rutin and quercetin in Swiss albino mice exposed to gamma radiation. *J. Med. Phys.* **2013**, *38*, 87.
38. Abeda, Z.H.; Sie, R.S.; Ayolie, K.; Yapo, S.E..; Coulibaly, S.; Kouassi, K.M.; Kouakou, T.H. Free Radical Scavenging Properties and Antioxidant Activities of Some Anthocyanins Purified from Roselle (Hibiscus sabdariffa L.) Callus Using In-Vitro Tests. *Res. J. Pharm. Biol. Chem. Sci.* **2015**, *6*, 320–329.
39. Wang, J.; Fang, X.; Ge, L.; Cao, F.; Zhao, L.; Wang, Z.; Xiao, W. Antitumor, antioxidant and anti-inflammatory activities of kaempferol and its corresponding glycosides and the enzymatic preparation of kaempferol. *PLoS One* **2018**, *13*.
40. Sun, Z.R.; Liu, H.R.; Hu, D.; Fan, M.S.; Wang, M.Y.; An, M.F.; Zhao, Y.L.; Xiang, Z.M.; Sheng, J. Ellagic Acid Exerts Beneficial Effects on Hyperuricemia by Inhibiting Xanthine Oxidase and NLRP3 Inflammasome Activation. *J. Agric. Food Chem.* **2021**, *69*, 12741–12752.
41. Chun, O.K.; Kim, D.O.; Lee, C.Y. Superoxide Radical Scavenging Activity of the Major Polyphenols in Fresh Plums. *J. Agric. Food Chem.* **2003**, *51*, 8067–8072.
42. Dargemont, C.; Kühn, L.C. Export of mRNA from microinjected nuclei of Xenopus laevis oocytes. *J. Cell Biol.* **1992**, *118*, 1–9.
43. Khalil, H.S.; Goltsov, A.; Langdon, S.P.; Harrison, D.J.; Bown, J.; Deeni, Y. Quantitative analysis of NRF2 pathway reveals key elements of the regulatory circuits underlying antioxidant response and proliferation of ovarian cancer cells. *J. Biotechnol.* **2015**, *202*, 12–30.
44. Mulvaney, K.M.; Matson, J.P.; Siesser, P.F.; Tamir, T.Y.; Goldfarb, D.; Jacobs, T.M.; Cloer, E.W.; Harrison, J.S.; Vaziri, C.; Cook, J.G.; et al. Identification and Characterization of MCM3 as a Kelch-like ECH-associated Protein 1 (KEAP1) Substrate. *J. Biol. Chem.* **2016**, *291*, 23719–23733.
45. Jain, A.K.; Bloom, D.A.; Jaiswal, A.K. Nuclear import and export signals in control of Nrf2. *J. Biol. Chem.* **2017**, *292*, 2052.
46. Bouton, C.; Demple, B. Nitric oxide-inducible expression of heme oxygenase-1 in human cells. Translation-independent stabilization of the mRNA and evidence for direct action of nitric oxide. *J. Biol. Chem.* **2000**, *275*, 32688–32693.
47. Ge, M.; Yao, W.; Yuan, D.; Zhou, S.; Chen, X.; Zhang, Y.; Li, H.; Xia, Z.; Hei, Z. Brg1-mediated Nrf2/HO-1 pathway activation alleviates hepatic ischemia–reperfusion injury. *Cell Death Dis. 2017 86* **2017**, *8*, e2841–e2841.
48. Murakami, Y.; Ito, M.; Ohsawa, I. Molecular hydrogen protects against oxidative stress-induced SH-SY5Y neuroblastoma cell death through the process of mitohormesis. *PLoS One* **2017**, *12*, e0176992.
49. Shen, Q.; Chada, S.; Whitney, C.; Newburger, P.E. Regulation of the Human Cellular Glutathione Peroxidase Gene During In Vitro Myeloid and Monocytic Differentiation. *Blood* **1994**, *84*, 3902–3908.

50. Fukai, T.; Siegfried, M.R.; Ushio-Fukai, M.; Griendling, K.K.; Harrison, D.G. Modulation of extracellular superoxide dismutase expression by angiotensin II and hypertension. *Circ. Res.* **1999**, *85*, 23–28.
51. Sen, P.; Chakraborty, P.K.; Raha, S. p38 Mitogen-activated protein kinase (p38MAPK) upregulates catalase levels in response to low dose H2O2 treatment through enhancement of mRNA stability. *FEBS Lett.* **2005**, *579*, 4402–4406.
52. Szychowski, K.A.; Rombel-Bryzek, A.; Dołhańczuk-Śródka, A.; Gmiński, J. Antiproliferative Effect of Elastin-Derived Peptide VGVAPG on SH-SY5Y Neuroblastoma Cells. *Neurotox. Res.* **2019**, *36*, 503–514.
53. CHEN, G.; PAN, S.F.; CUI, X.L.; LIU, L.H. Puerarin attenuates angiotensin II-induced cardiac fibroblast proliferation via the promotion of catalase activity and the inhibition of hydrogen peroxide-dependent Rac-1 activation. *Chin. J. Nat. Med.* **2018**, *16*, 41–52.
54. Hass, M.A.; Massaro, D. Regulation of the synthesis of superoxide dismutases in rat lungs during oxidant and hyperthermic stresses. *J. Biol. Chem.* **1988**, *263*, 776–781.
55. Bartnikas, T.B.; Gitlin, J.D. Mechanisms of biosynthesis of mammalian copper/zinc superoxide dismutase. *J. Biol. Chem.* **2003**, *278*, 33602–33608.
56. Xu, J.; Zhang, Y.; Ren, G.; Yang, R.; Chen, J.; Xiang, X.; Qin, H.; Chen, J. Inhibitory Effect of Delphinidin on Oxidative Stress Induced by H2O2 in HepG2 Cells. *Oxid. Med. Cell. Longev.* **2020**, *2020*.
57. Ding, X.; Jian, T.; Wu, Y.; Zuo, Y.; Li, J.; Lv, H.; Ma, L.; Ren, B.; Zhao, L.; Li, W.; et al. Ellagic acid ameliorates oxidative stress and insulin resistance in high glucose-treated HepG2 cells via miR-223/keap1-Nrf2 pathway. *Biomed. Pharmacother.* **2019**, *110*, 85–94.
58. Kumar, A.D.N.; Bevara, G.B.; Kaja, L.K.; Badana, A.K.; Malla, R.R. Protective effect of 3-O-methyl quercetin and kaempferol from Semecarpus anacardium against H2O2 induced cytotoxicity in lung and liver cells. *BMC Complement. Altern. Med.* **2016**, *16*.
59. Xu, Y.; Ke, H.; Li, Y.; Xie, L.; Su, H.; Xie, J.; Mo, J.; Chen, W. Malvidin-3- O-Glucoside from Blueberry Ameliorates Nonalcoholic Fatty Liver Disease by Regulating Transcription Factor EB-Mediated Lysosomal Function and Activating the Nrf2/ARE Signaling Pathway. *J. Agric. Food Chem.* **2021**, *69*, 4663–4673.
60. Gęgotek, A.; Rybałtowska-Kawałko, P.; Skrzydlewska, E. Rutin as a Mediator of Lipid Metabolism and Cellular Signaling Pathways Interactions in Fibroblasts Altered by UVA and UVB Radiation. *Oxid. Med. Cell. Longev.* **2017**, *2017*.
